# Production of Barbari Bread (Traditional Iranian Bread) Using Different Levels of Distillers Dried Grains with Solubles (DDGS) and Sodium Stearoyl Lactate (SSL)

**DOI:** 10.3390/foods7030031

**Published:** 2018-03-01

**Authors:** Shirin Pourafshar, Kurt A. Rosentrater, Padmanaban G. Krishnan

**Affiliations:** 1 Dairy and Food Science Department, South Dakota State University, Brookings, SD 57007, USA; SP8DS@hscmail.mcc.virginia.edu (S.P.); Padmanaban.Krishnan@sdstate.edu (P.G.K.); 2 Department of Agriculture and Biosystems Engineering, Iowa State University, Ames, IA 50011, USA; 3 Department of Food Science and Human Nutrition, Iowa State University, Ames, IA 50011, USA

**Keywords:** distillers dried grains with solubles, fortification, sodium stearoyl sactate

## Abstract

Bread is one of the oldest foods known throughout history and even though it is one of the principal types of staple around the world, it usually lacks enough nutrients, including protein and fiber. As such, fortification is one of the best solutions to overcome this problem. Thus, the objective this study was to examine the effect of three levels of distillers dried grains with solubles (DDGS) (0%, 10% and 20%) in conjunction with three levels of SSL (sodium stearoyl lactate) (0%, 2% and 5%) on physical and chemical properties of Barbari bread (traditional Iranian bread). To the best of our knowledge, this is the first study to evaluate DDGS and Sodium Stearoyl-2-Lactilate (SSL), as sources of fortification in Barbari bread. The results showed that incorporation of 20% of DDGS and 0% SSL caused a significant increase in the amount of fiber and protein. As for the physical attributes, using higher amount of DDGS caused a darker color, and as for the texture parameters, the highest firmness was measured when 10% DDGS and 5% of SSL were used. Different Mixolab and Rapid Visco Analyzer (RVA) parameters also were measured with varying results. The findings of this study show that DDGS can be a valuable source of fiber and protein, which can be used as a cost effective source to fortify cereal-based products.

## 1. Introduction

Cereals are the edible seeds or grains of the grass family, *Gramineae*. Because cereals are inexpensive and readily available, humans in almost every country have used them as major food staples for centuries. Cereals and cereal products are an important source of energy, protein and fiber [1]. Wheat is the most important cereal, and is commonly consumed worldwide. Historians do not know exactly where wheat was first cultivated, but sources point to either Syria-Palestine or southern parts of Anatolia. Wheat cultivation spread from Palestine to Egypt and then from northern Mesopotamia to Persia, where bread was first developed. From there, the growth of wheat and bread spread in all directions [2]. Although whole wheat as a food component has high nutritional value, a considerable proportion of the grain’s nutrients are lost during the milling processes. Thus, the importance of adding value to those products made with all-purpose flour or other wheat flours increases. Since bread is the most consumed cereal product, fortification can help combat problems such as malnutrition.

One way of fortifying cereal products, especially breads, may be through the use of distillers dried grains. Distillers dried grains with solubles (DDGS) is a co-product resulting from the fermentation of cereal grains, mostly corn, for the production of ethanol. As a result of increase in ethanol production, there is an increase supply of DDGS as well [3]. America’s corn farmers have improved their cultivation techniques significantly and increased their yield potential. In 1935, 82 million acres of corn were harvested in United States (U.S.) with an average yield of 24.2 bushels per acre; by 1950, the yield increased to 38.2 bushels per acre. Then in 1956, the problem with farms was the abundance of corn. Corn production continued increasing so that the yield was 149 bushels per acre by 2006. The increase in corn encouraged cattle feeding in the U.S, and with the growth of the ethanol industry, the demand for corn has improved [4]. Furthermore, researchers have identified the potential value of DDGS as a source of protein, which often ranges from 27% to 35%, fiber, minerals and vitamins [5]. As a result, scientists and engineers have been trying to find different ways of using DDGS in human foods, rather than solely as livestock feed. Researchers have explored incorporating DDGS in food products, especially cereal-based products. For instance, in a study done by Wu et al. [6], spaghetti was supplemented with corn distillers dried grains. Additionally, Finley and Hanamoto [7] used brewer’s spent grain in bread, while Tsen et al. [8,9] used DDG flour in the production of bread and cookies and then evaluated the physical and chemical properties of final products. Corn distiller’s grains have also been used in spaghetti [8]. Furthermore, the effect of DDGS on quality of cornbread has been investigated by Liu and colleagues [10].

Sodium Stearoyl-2-Lactilate (SSL) is an anionic emulsifier which is effective in increasing dough strength. Emulsifiers are mostly used in the baking industry to enhance baking quality. They can prevent mechanical damage to fermented dough, increase shelf life and improve the texture of baked products [11]. Hydrocolloids are often used in bread to improve the volume and texture, extend shelf life, and make softer bread crumbs [12]. SSL can function through interactions with flour protein which will improve the viscoelasticity of the dough [11]. Other protein-reactive softeners, such as DATEM (diacetyl tartaric ester of monoglycerides), can increase the strength of gluten protein matrix which will, in turn, improve loaf volume and tighten crumb structure [13].

Flat breads have a very short shelf life, usually a few hours. Because of that, many studies have been done to increase the shelf life of flat breads. For example, in a study by Qarooni [2], the anti-staling effect of ingredients such as shortening on Barbari bread’s quality was investigated. The results from that study showed that adding 0.5% SSL and 0.3% shortening made the bread edible for up to 36 h, instead of the normal 16 h. Different Middle Eastern breads are made with various types of flours. For example, round shaped Baladi and Aish Meharha from Egypt and Bazlama, Pide and Yufca from Turkey are mainly made with wheat flour. Morocco has pan fried bread made with semolina flour. Afghanistan and Tajikistan have Bolani bread, which is flat bread stuffed with different vegetables. Although these breads are mainly made with wheat and other types of flours, each of them has its own physical and chemical characteristics. However, they may have deficiencies in certain nutrient components that can be remedied through fortification.

Among Middle Easterners, Iranians consume four major types of breads: Barbari, Lavash, Sangak and Taftoon, with Barbari being the most popular. On average, Barbari crust has a thickness of 1–2 mm, length of 67–75 cm, and width of 13.5–20 cm. Traditionally, this bread is made with all-purpose flour and the final product contains about 11% protein, 10% fiber and 0.5% fat. Barbari is thick and oval shaped, and is often topped with poppy seeds. The golden color of this bread comes from Romal, a mixture made from flour, baking soda, and boiling water which is brushed on the dough before baking. Barbari has a special aroma and its taste depends on the amount of sour dough and baking time.

In this study, three levels of DDGS (0%, 10% and 20%) and three levels of SSL (0%, 2% and 5%) were used for substitution of wheat flour in Barbari bread. The objectives of this study were to understand (1) the impact of substitution of three levels of DDGS and SSL on the physical and chemical attributes of final bread products, and (2) to study the changes in the physical properties of the dough with different levels of substitutions of DDGS and SSL.

## 2. Materials and Methods

### 2.1. Experimental Design

Distillers dried grains with solubles (DDGS) was obtained from a commercial fuel ethanol plant in South Dakota. All-purpose wheat flour and other ingredients were purchased from local markets. A control sample of Barbari bread was baked at 550 °C for 10 min. To fortify this bread three levels of DDGS substitution (0%, 10% and 20%) and three levels of SSL substitution (0%, 2% and 5%) were used, producing a two-factor design each with three levels, having a full factorial design. Two loaves of breads for each level of substitution were baked. All properties of each of these loaves were analyzed using three replications; thus, *n* = 6 measurements for each property, and for each treatment combination. In total, 54 samples were analyzed. Table 1 shows the amount of all-purpose flour, DDGS and SSL to be used in samples.

### 2.2. Preparation

For the sour dough, 1 g of salt, 9 g of active dry yeast (Red Star Active Dry Yeast, purchased from local market), 400 g of flour (Gold Medal All-purpose Flour, purchased from local market) with 650 g of water were used and only 35 g of sour dough was used for each batch. Romal topping for the bread required 4.2 g of flour, 4.2 g of baking soda and 85 g of water.

For the control, bread was made with only wheat flour, 880 g of all-purpose flour (Gold Medal All-purpose Flour) was used; the rest of the ingredients were 4.2 g of sugar, 4.2 g of salt, and 689.76 g of water. For the other breads, the same ingredients were used, varying only the amount of flour.

### 2.3. Bread Production

Sour dough was prepared 18 h before bread preparation during which, the sour dough was covered and left at room temperature. In terms of bread preparation, first the yeast was dissolved in warm water and then sugar was added and put aside for 10 min. This was then mixed with salt and water and then flour was gradually added and sour dough from the previous day was added as well. The mixture was mixed until the dough was no longer sticky. The next step was proofing, where the dough was placed in a proofing chamber for an hour and half for further activity of the yeast. Then, 400 g of dough was weighed and kneaded to form a 20 inch (50.8 cm) by 20 inch (50.8 cm) square, using a square frame to assure consistency in dimension of all breads. The thickness of the dough was measured in three different areas at the edges, then the Romal was made and brushed on top of dough. The dough was put aside for 10 min. Next, bread was baked at 500 °C for 10 min on a pizza stone (14 inch by 16 inch) to make baking condition close to that of the traditional ovens used for Barbari in Iran.

The other breads were made the same way except for the amount of flour, DDGS and SSL which were incorporated accordingly in each bread sample (Table 1). In the breads other than control, the same procedure was used but with different proportions of all-purpose flour. The mixing time and other details of preparation were the same for control and all other breads.

### 2.4. Physical and Chemical Properties

A texture analyzer was used to study the firmness and extensibility of the bread samples using two different probes: SMS/Chen-Hoseney Dough stickiness RIG and Pizza Tensile RIG, (TX.XT-plus, Texture Technologies Corp., Scarsdale, NY, USA). For measurement of each of these variables, duplications were done for each loaf (*n* = 2), for a total of four samples for each type of bread, or 24 samples. The thickness at the edges and the center of the bread was measured using Vernier calipers (Digimatic Calipers w/Absolute Encoders, Series 500, Mitutoyo Corporation, Kawasaki, Kanagawa, Japan). For the center, because of the bubbles which were formed in the middle of breads, the thickness was measured three times. After that, breads were grinded and moisture content as well as water activity were determined. In order to determine the water activity, water activity meter for food quality was used (Aqua lab CX-2, Decagon Devices, Inc., Pullman, WA, USA). Color was measured by spectrophotometer (Minolta CM-508d, Ramsey, NJ, USA) in which L* is the measure of lightness, a* is the measure of greenness to redness, and b* is the measure of blueness to yellowness. The color was determined for the baked products just like it was done for the dough, and L*, a* and b* were measured to get the color values.

Protein content was measured using the American Association of Cereal Chemistry (AACC) method for combustion—AACC approved method 46-0 [14] with a CE Elantech instrument (Flash EA 1112, ThermoFinnigan Italia S.p.A., Rodano (MI) Italy). In this method, the amount of nitrogen which was determined by the machine was converted into protein using a conversion factor of 5.7. For the determination of neutral detergent fiber (NDF), AACC approved method 30-25 (2010) was used. Fat content was determined using AOAC method 920.39 (2003) with an automated extractor Soxhlet using petroleum ether (CH-9230, Buchi Laborotechnik AG, Flawil, Switzerland). Moisture content was determined using the AACC approved method 44-19 [15] convection oven drying at 135°C (Model Labline, Inc. Chicago, IL, USA).

The rheological properties of the dough were determined using a Mixolab (Tripette and Renaud Chopin, Villeneuve La Garenne cedex, France) and Rapid Visco Analyzer (RVA) (Newport Scientific Pty. Ltd., Warriewood, Australia). For the Mixolab, the minimum torque (C_2_), peak torque (C_3_), cooking stability (C_4_), set back (C_5_) and the α, β, and γ were evaluated. As for the RVA, peak viscosity, temperature at peak viscosity, time to peak viscosity, and breakdown were measured.

### 2.5. Data Analysis

All collected data were analyzed with Microsoft Excel v.2007 and SAS v.9.0 software (SAS Institute, Cary, NC, USA) using a Type I error rate (α) of 0.05, by analysis of variance (ANOVA) to identify significant differences among treatments. Post-hoc Fisher’s Least Significant Differences LSD tests were used to determine where the differences occurred.

## 3. Results and Discussion

The results for each measurement are summarized in following tables: Table 2 shows the effect of adding DDGS or SSL on chemical properties of final breads; Table 3 shows the effect of adding both DDGS and SSL on chemical properties of final breads; Table 4 shows the main effect of adding DDGS or SSL on physical properties of final breads; and Table 5 describes the effect of adding both DDGS and SSL on physical attributes of final breads. The results from treatment effects on Mixolab and RVA are shown in Table 6, Table 7, Table 8, Table 9 and Table 10, respectively. Table 11 and Table 12 show the results for the main effect and treatment effect of DDGS and SSL on bread quality, respectively.

### 3.1. Chemical Properties

#### 3.1.1. Protein

The protein content of bread can be influenced by the Maillard reaction and also the aggregation, which can happen due to the dehydration of the surface due to high temperature during baking. In this study, the results from the main effect of DDGS showed that the highest content of protein was obtained in the bread substituted with 20% DDGS, which was expected since DDGS is a rich source of protein. As for SSL, the highest protein value was for bread with 0% SSL, while the lowest was for the bread made with 5% SSL. Thus, in the treatment effects, the highest value for protein content was the bread made with 0% SSL and 20% DDGS; a significant difference was observed between breads made with this treatment and control. This result is in accordance with a study by Reddy et al. [16], which showed that addition of DDG in the production of muffins resulted in higher protein content compared to controls. Another study showed that the addition of DDGS to chocolate chip cookies resulted in an increased protein content by 30% [17]. The protein content of the bread can be the direct reflection of fermentation in dough, because fermentation is an important step for the protein solubilization [18].

#### 3.1.2. Fiber

As expected, the highest value of fiber was in the bread made with 20% DDGS and 0% of SSL, with a significant difference from the control bread. Up to 5% addition of SSL reduced fiber in the bread. DDGS is a very good source of dietary fiber, which can increase the nutritional value and rheological properties of bread. Although fiber can increase the water absorption through hydrogen binding which is due to the hydroxyl groups in the fiber structure, it can reduce bread volume and affect the texture as well [19]. Since the non-enzymatic browning among peptides can produce fiber-like substances, fiber content of Barbari can increase as a result of baking [20]. Our result is consistent with other studies which also showed that incorporation of DDGS in other products had similar results for fiber content. For instance, addition of corn distillers (CDS) into spaghetti resulted in enhancement of fiber content up to 12–14% in comparison with the control sample [8]. In another study, it was shown that addition of DDGS in chocolate chip cookies and banana bread increased the fiber content of final products [17].

#### 3.1.3. Fat

Addition of DDGS at level of 20% resulted in the highest value of fat for the DDGS main effect, and addition of 5% SSL resulted in the highest fat content for the SSL main effect. For the treatment effect combinations, addition of 20% DDGS and 5% SSL, resulted in a significantly higher value of fat content compared to the control bread. According to Rasco et al. [17], this increase can be due to the high fat content of DDGS. Addition of SSL to the bread formulation can help in evenly distribution of lipids, and generation of fatty acid-amylose complex which may remain in the starch granules [21]. Similar results can be observed in other studies. For example, in a study by Reddy et al. [16], the amount of lipid in soft wheat DDG increased up to 1.4–2.4 times when compared to whole grain wheat prior to fermentation; however, incorporation of 10% DDG into wheat muffin did not change the fat content significantly.

#### 3.1.4. Moisture

In general, Barbari bread has the highest moisture content among Iranian breads [20]. However, in this study, the highest moisture content as a main effect of SSL was found in the bread without SSL, and for the main effect of DDGS, the highest value was for the bread made without any DDGS. Although in a study, it was shown that addition of DDGS to baked products resulted in an increase in the water absorption [22], in our study, no significant differences were seen between different treatments in moisture content, as they had almost the same amount of moisture.

### 3.2. Physical Properties

#### 3.2.1. Thickness of Bread

Addition of 20% DDGS incorporated with 2% SSL resulted in the highest value of thickness, likely due to the use of SSL in the bread. The lowest value was for control, which was significantly lower compared to bread made with 20% DDGS and 2% SSL. Thickness can be affected by both the fiber content and the use of hydrocolloids. Dietary fiber, in general, reduces the volume of bread. In a study by Park et al. [23], incorporation of fiber into bread resulted in decrease of volume by 5–15% due to the poor gas retention in bread. Also, another study showed that supplementation of 15 and 25% DDGS reduced the average thickness of cookies [24]. On the other hand, using hydrocolloids in bread formulation, such as SSL, can improve the formations of gluten networks and increase the volume of the bread.

#### 3.2.2. Texture

Texture has a direct impact on consumer acceptability and it has other effects through releasing flavor and its influence on appearance [9]. In this study, two textural attributes of final bread samples were measured; firmness and extensibility. The highest value of extensibility was found in bread made with 0% and 2% SSL, and increasing the value of SSL up to 5% resulted in the lowest amount of extensibility. Also, addition of DDGS up to 20% resulted in decreased extensibility which occurred due to the low amount of gluten content in bread. Thus, the highest value of extensibility was measured in the bread made with 0% DDGS and 2% SSL, and the lowest extensibility value was for the bread made with 20% DDGS and 5% SSL which were significantly different than each other. The most important factor which gives extensibility to bread is the gluten and SSL can help strengthen the gluten network and retain gas which is produced by yeast in the dough [21].

One important factor which can result in an increased firmness of bread is the amount of fiber in the flour. In one study, bread with 2% fiber-supplementation had significantly firmer crumb compared to control bread. This was due to the thickening of walls surrounding the air bubbles in the crumb [19]. The highest amount of firmness, for addition of DDGS was the bread made with 10% DDGS, but it was not significantly different from the 20% DDGS. As for the addition and main effect of SSL, the highest amount of firmness was determined in the bread made with 5% SSL. As for the treatment effect, the treatment with 10% DDGS and 5% SSL had the highest amount of firmness and was significantly different from the control bread. However, in a study by Marco and Rosell [25], addition of hydroxypropyl methylcellulose (HPMC) in bread resulted in a significant decrease in crumb hardness; but addition of protein increased the hardness. Thus, another factor, which can improve bread firmness, is the protein content. In a study by Ahlborn et al. [26] it was shown that the force required to compress bread was higher for the bread with protein added than standard wheat bread. The protein in bread is gluten, which provides unique functional properties in bread and is responsible for the protein-starch interaction, providing specific viscoelastic properties in bread dough [26]. Thus, with the high content of fiber and protein as well as the effect of SSL on gluten formation, it can be implied that both DDGS and SSL affected the firmness and texture of bread samples.

#### 3.2.3. Water Activity

There were no significant differences in water activity between different treatments, but the treatment with 20% DDGS and 0% SSL was slightly higher compared to the other treatments. In a study by Marco and Rosell [25], addition of HPMC resulted in a significant decrease of moisture due to hydrocolloids retaining water. Water has an important role in the production of bread; it takes part in starch gelatinization, protein denaturation, flavor, and color development. Fiber plays a role in the water absorption; as the amount of fiber increases, the flour-water absorption increases as well [27].

#### 3.2.4. Color

Color is a quality parameter which affects consumer acceptability of bread. The formation of the golden yellow color on Barbari bread is produced because of the Romal which is brushed on the bread. Romal is made from baking soda and wheat flour dissolved in warm water, which leads to the formation of dextrin during baking and finally the golden color. Thus, the type of flour and the browning reaction which are non-enzymatic reactions, play important roles. Since bread contains both reducing sugars and amino groups, when it is heated, caramelization and Maillard reaction may take place at the same time. One study showed that in order for browning reactions to take place, temperature greater than 120 °C and water activity less than 0.6 are required [28]. In this study, L*, a* and b* were determined for bread samples. In terms of DDGS main effect, addition of 20% DDGS had the highest L* value which is due to the natural dark color of DDGS. For SSL main effect, there were no significant differences between different values of SSL. Overall, bread made with 20% DDGS and 2% SSL had the highest value of L* which was significantly different than breads made with 0% DDGS, 2 and 5% of SSL. This was due to the incorporation of SSL in the flour matrix and Maillard reactions during baking which contributes to the golden and brown color of breads. The a* value was highest for the bread made with 10% DDGS, so it was more red than all others; however, there were really no significant differences for different amounts of DDGS in formulations. As for SSL, the highest value was for no addition of SSL. The treatment effect showed the highest value was for the control bread, but there were no significant differences between different treatments. The highest b* value was in the bread made with 20% DDGS and 5% SSL, which resulted in yellower bread, while the b* value for the control bread and breads made with 20% DDGS, 2 and 5% SSL had no significant differences. In a study by Rasco et al. [17], it was shown that 30% blends of all-purpose flour and DDGS had a darker color, more red and more yellow than control blends. Color also can be affected by the raw ingredients, especially flour. DDGS is an ingredient which can have a great impact on the final product’s color. Different bakery products have been tested and made with DDGS. For example, adding 30% DDGS to muffins made them darker in comparison to control muffins, and adding up to 20% DDGS resulted in a darker color in hush puppies [29].

#### 3.2.5. Quality

In this study, Barbari breads were also tested for quality attributes such as uniformity, thickness, softness, size and color by the test panelists. Bread made with 10% DDGS and 0% SSL had the highest uniformity, and was significantly different than control bread. Other studies show that adding up to 10% DDG to wheat muffins changed neither appearance nor texture; however, a grainy texture in muffins supplemented with 20% DDG was found [16]. In another study, adding 30% DDG to muffins made the muffins dry and more irregular in cell distribution compared to the control [29]. As for the size, the highest amount was related to the bread made with 0% of DDGS and 2% of SSL but it was not significantly different than the control; the lowest was determined for the bread made with 10% DDGS and 0% SSL. In evaluation of bread thickness, bread made with 20% DDGS and 5% SSL had the highest amount, while the one made with 10% DDGS and 5% SSL had the lowest thickness.

**Figure 1:**
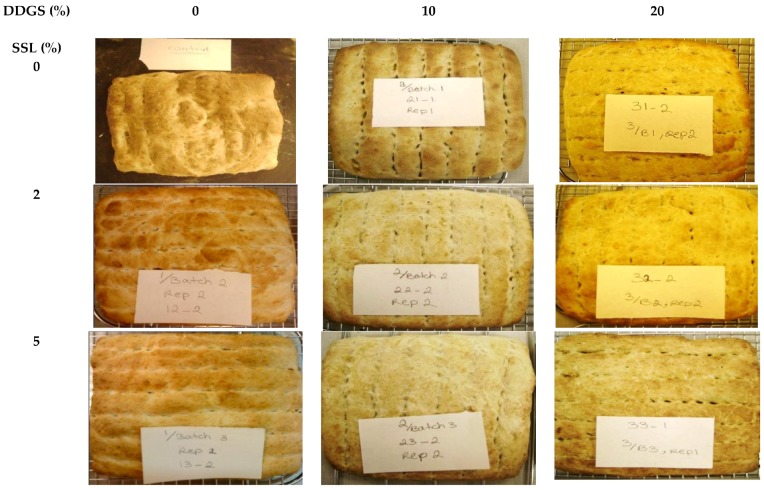
Final bread products baked with different blends of distillers dried grains with solubles (DDGS) and sodium stearoyl-2-lactate (SSL).

The results for softness of samples showed that bread made with 0% DDGS and 5% SSL was softest. Addition of emulsifiers to baked products can lead to crumb softening because of the interaction of emulsifiers with the starch. Also, by preventing amylose and amylopectin retrogradation, they can inhibit bread staling [27]. Finally, in evaluation of color (Figure 1), bread made with 0% DDGS and 2% SSL had a better color which was significantly different than the control (0% DDGS and 0% SSL). In general, as DDGS increased, color decreased.

### 3.3. Mixolab Results

As shown in Table 8, different Mixolab parameters were measured in this study. Development time (C1) was a maximum value when 10% DDGS and 5% SSL were used, this part of the curve is where water is being added to the flour and the starch granules and proteins start to absorb water, making them swelled; C1 values were close to each other, and there were no significant differences between different levels of DDGS and SSL which indicates resistance of the dough in all treatments. The measurement of minimum torque (C2), which shows the protein behavior, also didn’t show significant differences between different treatment effects. This part of the curve shows the protein behavior and how strong the protein can be in the dough network. Since addition of DDGS added to the protein value of the dough, it is reasonable to see insignificant differences in the C2 values. As for peak torque (C3), the highest value was for control sample, which indicates better quality of starch and shows a decrease as DDGS increased. The cooking stability (C4), which is the indication of amylastic activity, had the highest value for 0% DDGS and 5% SSL, and the lowest value was measured when 20% DDGS and 5% SSL were used, with significant differences between the two treatments. The set back (C5) had the highest value for 10% DDGS and 2% SSL, and it had a significant difference in treatment with lowest value of C5 in which 10% DDGS and 5% SSL were used; in this region retrogradation of starch happens, which caused an increase in the consistency as the temperature was decreasing.

Other parameters determined for Mixolab measurement were *α*, with the highest value for treatment with 20% DDGS and 5% SSL, which indicated that addition of DDGS to the wheat flour can help improving the protein network of the dough, as *α* shows the protein break down in the dough matrix. β, which is a good indicator of starch gelatinization, had the highest value for the treatment with 10% DDGS and 2% SSL, and lowest value for 0% of DDGS and 5% SSL, with significant differences between them; and γ, which shows the rate at which cooking stability is reached, had the highest value in the treatment with 0% DDGS and 5% SSL. Water absorption in the developing dough had its highest value for the treatment of 0% DDGS and 2% SSL, with significant difference from that of control dough. Also, the stability time had its highest value for the control sample, but the lowest value for the incorporation of 5% SSL and 0% DDGS. Stronger wheat flours have the ability to absorb and retain more water compared to weak flours (flours with lower protein content). Higher development time indicates stronger flour (flours with higher protein content) [12]. These results are in accordance with other studies; for instance, using an amylograph, it was determined that the addition of SSL to defatted soybean meal and defatted sesame meal caused slight delay of on-set of starch gelatinization and the flour with higher SSL had higher water absorption [30]. In another study, addition of surfactants decreased water absorption of flour by about 0.4–1.2%, and the dough development time decreased by 0.5 min with SSL and DATEM (mono and diacetyltartaric acid) combination [31]. In the study by Tsen et al. [9], replacement of flour by 10 to 20% DDG reduced dough development time.

### 3.4. Rapid Visco Analyzer (RVA) Results

RVA can be used to measure pasting properties. The RVA pasting curve can detect differences in flour viscosity with a small amount of sample and in a short period of time [32]. Because in our study, SSL was used, peak viscosity, pasting temperature and setback measured by the RVA can be useful in predicting the dough behavior during baking. Wheat chemistry is complex, involving interactions between gliadin and glutenin as well as starch and gluten network. Interactions between gliadin and glutenin are responsible for the initial RVA viscosity [33], and these interactions can affect bread quality. In our study, the highest peak value occurred for the treatment in which 0% DDGS and 5% SSL was used, which was significantly different from the sample made with 20% DDGS and 5% SSL. When starch granules start swelling, viscosity of the paste will increase until a maximum viscosity is reached at “peak viscosity” which is the reflection of water binding capacity of the starch [34]. The trough value was the highest when 0% of DDGS and 5% of SSL were used compared to the control sample which had the lowest value. When 20% DDGS and 5% of SSL were used, the break down had its lowest value compared to the control sample with highest value. In one study, the behavior of wheat flour subjected to shear stress was affected by adding hydrocolloids, causing an increase in water absorption and dough development time [12]. The final viscosity had the highest value with incorporation of 5% SSL and 0% DDGS which were significantly different from the control. As for the setback time, the highest value was measured when 0% DDGS were used and 5% SSL. The control sample had the lowest peak time, and the highest peak time value occurred when 0% DDGS and 5% SSL were used, with significant differences between these two treatments. The last parameter measured was pasting temperature, with the highest value when 0% DDGS and 5% SSL were used. This was expected because of the role of SSL in interaction between starch and gluten network. Changes in the gluten network, such as conformational modifications and loss of hydrogen bonds can decrease RVA viscosity during heating [33]. On the other hand, higher swelling capacity of starch will result in a higher peak viscosity, as the ability of the starch to withstand high temperatures and shear stresses is important [35].

## 4. Conclusions

Flat breads made from various types of flours, especially wheat flour, are the oldest type of food. Certain deficiencies and nutritional problems exist with cereal-based products, particularly breads. Because of the high demand for flat breads in most countries, fortification of breads can help providing additional nutrients to consumers. DDGS is a good source of protein as well as fiber and can be an inexpensive source for fortification of flat breads. In this study, three levels (0%, 10% and 20%) of DDGS were added to the wheat flour to study the changes in the physical and chemical properties of the final bread products. In addition to the DDGS, SSL was also added to the formulation at three levels (0%, 2% and 5%). The resulting breads were measured for their physical and chemical properties. Overall, the results of this study showed that addition of DDGS can increase fiber and protein values of Barbari bread significantly, while addition of 5% SSL can lead to a softer texture of bread. As the DDGS content increased in the bread formulations, the L* increased because of the darkness in the DDGS. In addition to the physical and chemical properties of the final products, the rheological properties of the flours were also measured using Mixolab and RVA. The results showed that water absorption of the developing dough had its highest value for the treatment of 0% DDGS and 2% of SSL. For the RVA, highest amount of peak value was for the treatment in which 0% of DDGS and 5% of SSL. The final viscosity had the highest value with incorporation of 5% SSL and 0% of DDGS.

## Figures and Tables

**Table 1:** Experimental design ^1^.

Treatment	Wheat ^2^ (%)	DDGS ^3^ (%)	SSL ^4^ (%)
**0 (Control)**	**100**	**0**	**0**
1	98	0	2
2	95	0	5
3	90	10	0
4	88	10	2
5	85	10	5
6	80	20	0
7	78	20	2
8	75	20	5

^1^ Each treatment was replicated twice. ^2^ From market source, Hy-Vee bleached all-purpose flour. ^3^ DDGS is distillers dried grains with solubles, obtained from a commercial fuel ethanol plant. ^4^ SSL is sodium stearoyl-2-lactate.

**Table 2:** Main effects on chemical properties of baked breads ^1^.

Effect (% Substitution)	Protein (% db) ^2^		Fiber (% db)		Fat (% db)		Moisture	
	Mean	SD ³	Mean	SD	Mean	SD	Mean	SD
DDGS (%)								
0	11.10 ^a^	0.23	0.45 ^c^	0.37	1.22 ^c^	0.84	0.43 ^ab^	0.03
10	12.48 ^b^	0.15	2.05 ^b^	0.37	1.75 ^b^	1.09	0.40 ^b^	0.35
20	13.66 ^c^	0.10	3.71 ^a^	0.42	2.4 ^a^	1.12	0.38 ^a^	0.57
SSL (%)								
0	12.51 ^a^	1.17	2.42 ^a^	1.44	0.86 ^c^	0.40	0.44 ^a^	0.04
2	12.49 ^a^	1.04	2.04 ^b^	1.59	1.49 ^b^	0.59	0.42 ^a^	0.06
5	12.24 ^b^	1.21	1.75 ^b^	1.38	3.07 ^a^	0.68	0.42 ^a^	0.04

^1^ Different letters for a given dependent variable denote significant differences (α = 0.05) across treatment conditions for that independent variable. ^2^ All properties are reported as % dry basis (db). ^3^ SD is standard deviation.

**Table 3:** Treatment effects on chemical properties of baked breads ^1^.

DDGS (%)	SSL (%)	Protein (% db) ^2^		Fiber (% db)		Fat (% db)		Moisture (% db)	
		Mean	SD ^3^	Mean	SD	Mean	SD	Mean	SD
0	0	11.17 ^d^	0.04	0.90 ^d^	0.04	0.50 ^f^	0.00	0.45 ^a^	0.02
0	2	11.29 ^d^	0.24	0.28 ^e^	0.25	0.87 ^e^	0.03	0.43 ^ab^	0.03
0	5	10.86 ^e^	0.12	0.17 ^e^	0.05	2.29 ^c^	0.10	0.40 ^ab^	0.01
10	0	12.58 ^b^	0.05	2.26 ^c^	0.36	0.73 ^ef^	0.05	0.41 ^ab^	0.00
10	2	12.58 ^b^	0.01	2.02 ^c^	0.05	1.43 ^d^	0.01	0.38 ^b^	0.01
10	5	12.29 ^c^	0.07	1.88 ^c^	0.64	3.11 ^b^	0.06	0.41 ^ab^	0.07
20	0	13.79 ^a^	0.05	4.09 ^a^	0.21	1.35 ^d^	0.20	0.47 ^a^	0.05
20	2	13.62 ^a^	0.02	3.84 ^a^	0.00	2.19 ^c^	0.13	0.46 ^a^	0.08
20	5	13.57 ^a^	0.056	3.20 ^b^	0.07	3.82 ^a^	0.15	0.46 ^a^	0.03

^1^ Different letters for a given dependent variable denote significant differences (α = 0.05) across treatment conditions for that independent variable. ^2^ All properties are reported as % dry basis. ^3^ SD is standard deviation.

**Table 4:** Main effects on physical properties of baked breads ^1^.

Effect (% Substitution)	Firmness (N) ^2^		Extensibility (N) ^2^		Thickness (mm)		a_w_		L*		a*		b*	
	Mean	SD ^3^	Mean	SD	Mean	SD	Mean	SD	Mean	SD	Mean	SD	Mean	SD
DDGS (%)														
0	31.28 ^b^	8.98	19.06 ^a^	8.08	13.97 ^b^	3.24	0.93 ^a^	0.01	61.6 ^b^	5.88	9.78 ^a^	3.32	20.02 ^a^	4.67
10	42.62 ^a^	8.13	13.15 ^b^	6.14	14.80 ^ab^	1.01	0.88 ^a^	0.16	72.94 ^a^	2.68	10.22 ^a^	2.79	22.86 ^a^	2.15
20	39.10 ^a^	5.41	10.41 ^b^	2.99	16.06 ^a^	1.84	0.94 ^a^	0.00	71.03 ^a^	4.01	9.74 ^a^	1.57	23.67 ^a^	1.84
SSL (%)														
0	33.57 ^b^	10.91	17.65 ^a^	4.60	13.32 ^b^	2.86	0.94 ^a^	0.01	69.51 ^a^	4.77	11.01 ^a^	2.55	23.27 ^a^	1.71
2	38.85 ^ab^	5.12	15.55 ^a^	9.32	15.67 ^a^	1.46	0.89 ^a^	0.17	67.95 ^a^	9.39	8.63 ^a^	2.35	20.60 ^a^	4.85
5	40.57 ^a^	8.68	9.42 ^b^	2.56	15.85 ^a^	1.64	0.93 ^a^	0.013	68.13 ^a^	5.78	10.11 ^a^	2.43	22.68 ^a^	2.73

^1^ Different letters for a given dependent variable denote significant differences (α = 0.05) across treatment conditions for that independent variable. ^2^ The force was measured over time for firmness, and over travel distance for extensibility. ^3^ SD is standard deviation. a_w_ is water activity; L* is the measure of lightness; a* is the measure of greenness to redness; and b* is the measure of blueness to yellowness.

**Table 5:** Treatment effects on physical properties of baked breads ^1^.

DDGS (%)	SSL (%)	Firmness (N) ^2^		Extensibility (N) ^2^		Thickness (mm)		a_w_		L*		a*		b*	
		Mean	SD ^3^	Mean	SD	Mean	SD	Mean	SD	Mean	SD	Mean	SD	Mean	SD
0	0	20.95 ^d^	5.83	18.53 ^b^	5.28	9.70 ^d^	0.97	0.94 ^a^	0.00	65.14 ^cb^	6.56	13.54 ^a^	2.02	25.13 ^a^	1.27
0	2	38.75 ^bc^	5.13	27.05 ^a^	7.03	15.72 ^abc^	0.69	0.94 ^a^	0.00	55.97 ^d^	2.34	6.70 ^b^	1.71	14.99 ^c^	2.19
0	5	34.15 ^c^	2.83	11.59 ^cd^	1.49	16.49 ^ab^	0.56	0.92 ^b^	0.00	63.74 ^cd^	5.21	9.11 ^ab^	0.28	19.95 ^b^	0.11
10	0	42.36 ^bc^	4.50	21.05 ^b^	2.71	14.72 ^bc^	0.98	0.93 ^a^	0.00	70.97 ^abc^	3.33	11.24 ^ab^	0.26	23.02 ^ab^	0.85
10	2	35.19 ^bc^	3.89	9.18 ^cd^	1.51	0.38 ^c^	0.01	0.92 ^b^	1.30	74.62 ^a^	1.44	8.98 ^ab^	3.18	22.21 ^ab^	2.98
10	5	50.33 ^a^	7.34	9.23 ^cd^	1.97	15.24 ^abc^	0.79	0.93 ^a^	0.02	73.23 ^ab^	3.04	10.45 ^ab^	4.85	23.35 ^ab^	3.50
20	0	37.42 ^bc^	6.85	13.38 ^c^	1.25	15.55 ^abc^	1.22	0.95 ^a^	0.00	72.41 ^abc^	0.75	8.25 ^b^	0.67	21.68 ^ab^	0.57
20	2	42.63 ^b^	4.20	10.43 ^cd^	1.23	16.83 ^a^	1.36	0.93 ^a^	0.00	73.28 ^ab^	1.01	10.21 ^ab^	1.41	24.61 ^a^	1.95
20	5	37.24 ^bc^	4.21	7.44 ^d^	2.51	15.80 ^abc^	2.81	0.94 ^a^	0.00	67.41 ^abc^	6.24	10.76 ^ab^	1.73	24.73 ^a^	0.94

^1^ Different letters for a given dependent variable denote significant differences (α = 0.05) across treatment conditions for that independent variable. ^2^ The force was measured over time for firmness and over travel distance for extensibility. ^3^ SD is standard deviation. a_w_ is water activity; L* is the measure of lightness; a* is the measure of greenness to redness; and b* is the measure of blueness to yellowness.

**Table 6:** Main effect of DDGS on Mixolab operational parameters ^1^.

Effect (% Substitution)	C1 Time (min)		C1 Torque (Nm)		C2 Torque (Nm)		C3 Torque (Nm)		C4 Torque (Nm)		C5 Torque (Nm)		α ^4^ (N-m/min)		β ^4^ (N-m/min)		γ ^4^ (N-m/min)		Water Abs ^3^ (%)		Stability (s)	
	Mean	SD ^2^	Mean	SD	Mean	SD	Mean	SD	Mean	SD	Mean	SD	Mean	SD	Mean	SD	Mean	SD	Mean	SD	Mean	SD
0	2.15 ^a^	2.23	1.09 ^b^	0.03	0.37 ^a^	0.06	1.49 ^a^	0.52	1.66 ^a^	0.35	2.34 ^a^	0.49	−0.05 ^a^	0.03	0.31 ^ab^	0.26	−0.01 ^a^	0.08	53.36 ^a^	2.88	7.60 ^a^	4.12
10	2.47 ^a^	2.29	1.12 ^ab^	0.03	0.36 ^a^	0.05	1.16 ^a^	0.46	1.29 ^a^	0.75	2.23 ^a^	0.95	−0.06 ^a^	0.03	0.37 ^a^	0.31	−0.01 ^a^	0.06	53.13 ^a^	2.05	5.77 ^a^	3.90
20	3.90 ^a^	3.48	1.16 ^a^	0.07	0.39 ^a^	0.06	0.69 ^b^	0.15	1.01 ^a^	0.67	2.20 ^a^	0.94	−0.07 ^a^	0.03	0.093 ^b^	0.034	0.02 ^a^	0.02	52.08 ^a^	2.12	5.80 ^a^	3.70

^1^ Different letters for a given dependent variable denote significant differences (α = 0.05) across treatment conditions for that independent variable. ^2^ SD is standard deviation. ^3^ Abs is absorption. ^4^ α shows protein breakdown, β shows gelatinization and γ shows cooking stability rate.

**Table 7:** Main effect of SSL on Mixolab operational parameters ^1^.

Effect (% Substitution)	C1 Time (Min)		C1 Torque (Nm)		C2 Torque (Nm)		C3 Torque (Nm)		C4 Torque (Nm)		C5 Torque (Nm)		α ^4^ (N-m/min)		β ^4^ (N-m/min)		γ 4 (N-m/min)		Water Abs ^3^ (%)		Stability (s)	
	Mean	SD ^2^	Mean	SD	Mean	SD	Mean	SD	Mean	SD	Mean	SD	Mean	SD	Mean	SD	Mean	SD	Mean	SD	Mean	SD
0	4.33 ^a^	2.34	1.13 ^a^	0.08	0.35 ^b^	0.03	1.31 ^a^	0.54	1.52 ^a^	0.11	2.27 ^a^	0.30	−0.08 ^b^	0.04	0.33 ^a^	0.20	0.00 ^a^	0.04	53.13 ^a^	2.41	10.22 ^a^	1.11
2	3.17 ^ab^	3.48	1.11 ^a^	0.05	0.42 ^ab^	0.05	1.20 ^ab^	0.52	1.51 ^a^	0.54	2.61 ^a^	0.43	−0.06 ^ab^	0.02	0.37 ^a^	0.32	0.00 ^a^	0.06	53.45 ^a^	2.71	5.74 ^b^	3.49
5	1.02 ^b^	0.14	1.13 ^a^	0.03	0.35 ^a^	0.06	0.83 ^b^	0.41	0.93 ^a^	0.90	1.89 ^a^	1.20	−0.04 ^a^	0.02	0.07 ^b^	0.07	0.00 ^a^	0.08	52.00 ^a^	1.89	3.20 ^b^	2.20

^1^ Different letters for a given dependent variable denote significant differences (α = 0.05) across treatment conditions for that independent variable. ^2^ SD is standard deviation. ^3^ Abs is absorption. ^4^ α shows protein breakdown, β shows gelatinization and γ shows cooking stability rate.

**Table 8:** Treatment effects on Mixolab operational parameters ^1^.

DDGS (%)	SSL (%)	C1 Time (Min)		C1 Torque (Nm)		C2 Torque (Nm)		C3 Torque (Nm)		C4 Torque (Nm)		C5 Torque (Nm)		α ^3^ (N-m/min)		β ^3^ (N-m/min)		γ ^3^ (N-m/min)		Water Abs ^3^ (%)		Stability (s)	
		Mean	SD ^2^	Mean	SD	Mean	SD	Mean	SD	Mean	SD	Mean	SD	Mean	SD	Mean	SD	Mean	SD	Mean	SD	Mean	SD
0	0	1.43 ^ab^	0.12	1.08 ^a^	0.00	0.37 ^ab^	0.04	1.73 ^a^	0.14	1.44 ^bc^	0.09	2.00 ^ab^	0.13	−0.04 ^abc^	0.04	0.50 ^ab^	0.01	−0.05 ^bc^	0.01	50.75 ^b^	2.05	10.55 ^a^	1.45
0	2	3.99 ^ab^	3.83	1.06 ^a^	0.01	0.37 ^ab^	0.05	1.71 ^a^	0.13	1.44 ^bc^	0.16	2.13 ^ab^	0.36	−0.08 ^abc^	0.03	0.47 ^abc^	0.04	−0.05 ^bc^	0.07	56.05 ^a^	1.48	9.81 ^a^	0.04
0	5	1.05 ^ab^	0.02	1.14 ^a^	0.02	0.37 ^ab^	0.13	1.04 ^abc^	0.86	2.10 ^a^	0.05	2.91 ^ab^	0.33	−0.03 ^ab^	0.01	−0.02 ^d^	0.01	0.06 ^a^	0.08	53.30 ^ab^	2.68	2.46 ^b^	1.75
10	0	5.42 ^ab^	0.43	1.12 ^a^	0.03	0.33 ^b^	0.02	1.59 ^ab^	0.15	1.53 ^abc^	0.14	2.35 ^ab^	0.39	−0.10 ^bc^	0.02	0.42 ^abc^	0.06	−0.01 ^abc^	0.00	54.65 ^ab^	2.33	10.21 ^a^	1.18
10	2	0.93 ^b^	0.02	1.11 ^a^	0.00	0.43 ^ab^	0.03	1.14 ^abc^	0.59	1.99 ^ab^	0.16	3.01 ^a^	0.12	−0.04 ^ab^	0.01	0.57 ^a^	0.51	0.04 ^a^	0.00	52.75 ^ab^	2.47	3.70 ^b^	2.44
10	5	1.07 ^ab^	0.21	1.12 ^a^	0.05	0.32 ^b^	0.02	0.76 ^bc^	0.02	0.37 ^d^	0.00	1.34 ^b^	1.25	−0.04 ^ab^	0.04	0.12 ^bcd^	0.01	−0.08 ^c^	0.05	52.00 ^ab^	1.41	3.40 ^b^	3.08
20	0	6.15 ^a^	1.24	1.18 ^a^	0.16	0.34 ^ab^	0.00	0.62 ^c^	0.09	1.60 ^abc^	0.09	2.46 ^ab^	0.26	−0.11 ^c^	0.04	0.07 ^cd^	0.01	0.05 ^a^	0.00	54.00 ^ab^	1.41	9.92 ^a^	1.52
20	2	4.59 ^ab^	5.55	1.17 ^a^	0.04	0.46 ^a^	0.04	0.76 ^c^	0.30	1.10 ^c^	0.78	2.70 ^ab^	0.04	−0.06 ^abc^	0.00	0.09 ^cd^	0.06	0.02 ^ab^	0.03	51.55 ^ab^	2.61	3.73 ^b^	2.33
20	5	0.96 ^b^	0.19	1.14 ^a^	0.00	0.37 ^ab^	0.00	0.69 ^c^	0.06	0.33 ^d^	0.09	1.44 ^ab^	1.59	−0.03 ^a^	0.00	0.11 ^bcd^	0.00	0.00 ^abc^	0.01	50.7 ^b^	1.41	3.75 ^b^	3.13

^1^ Different letters for a given dependent variable denote significant differences (α = 0.05) across all treatment conditions. ^2^ SD is standard deviation. ^3^ α shows protein breakdown, β shows gelatinization and γ shows cooking stability rate, Abs is Absorption.

**Table 9:** Main effects on RVA operational parameters ^1^.

Effects (% Substitution)	Peak 1 (cP)		Trough 1 (Nm)		Break Down		Final Visc ^3^ (cP)		Setback (cP)		Peak Time (min)		Pasting Temp (°C)	
	Mean	SD ^2^	Mean	SD	Mean	SD	Mean	SD	Mean	SD	Mean	SD	Mean	SD
DDGS (%)														
0	1532.0 ^a^	309.27	839.33 ^a^	360.90	692.66 ^a^	91.41	3243.83 ^a^	1968.38	2404.50 ^a^	1648.26	5.93 ^a^	0.56	86.13 ^a^	10.18
10	1208.17 ^b^	133.12	613.33 ^b^	181.97	594.83 ^b^	56.79	2152.50 ^b^	959.03	1539.17 ^b^	785.28	5.53 ^b^	0.29	85.21 ^a^	8.59
20	1017.50 ^b^	72.70	548.83 ^b^	23.49	468.66 ^c^	65.91	1561.00 ^b^	322.52	1012.17 ^b^	317.90	5.33 ^b^	0.04	88.36 ^a^	1.77
SSL (%)														
0	1121.67 ^b^	71.48	474.33 ^b^	59.53	647.33 ^a^	98.98	993.50 ^b^	250.70	519.16 ^b^	233.97	5.25 ^b^	0.05	79.15 ^b^	8.99
2	1233.33 ^ab^	221.02	661.33 ^ab^	163.97	572.00 ^b^	106.28	2676.50 ^a^	1089.27	2015.17 ^a^	935.38	5.65 ^a^	0.35	89.30 ^a^	0.75
5	1402.67 ^a^	423.11	865.83 ^a^	311.94	536.83 ^b^	132.10	3287.33 ^a^	1418.14	2421.50 ^a^	1132.29	5.88 ^a^	0.51	91.25 ^a^	2.02

^1^ Different letters for a given dependent variable denote significant differences (α = 0.05) across treatment conditions for that independent variable. ^2^ SD is standard deviation. ^3^ Visc is viscosity. RVA is Rapid Visco Analyzer.

**Table 10:** Treatment effects on RVA operational parameters ^1^.

DDGS (%)	SSL (%)	Peak 1(cP)		Trough 1 (Nm)		Break Down		Final Visc ^3^ (cP)		Setback (cP)		Peak Time (min)		Pasting Temp (°C)	
		Mean	SD ^2^	Mean	SD	Mean	SD	Mean	SD	Mean	SD	Mean	SD	Mean	SD
0	0	1205.50 ^d^	16.26	455.50 ^c^	0.70	750.00 ^a^	15.55	802.50 ^f^	427.79	347.00 ^e^	428.50	5.26 ^e^	0.00	75.00 ^b^	11.31
0	2	1501.50 ^b^	28.99	842.00 ^b^	171.11	659.50 ^ab^	142.12	3965.00 ^b^	486.48	3123.00 ^a^	315.36	6.06 ^b^	0.18	89.65 ^a^	0.91
0	5	1889.00 ^a^	84.85	1220.50 ^a^	191.62	668.50 ^ab^	106.77	4964.00 ^a^	272.94	3743.50 ^a^	464.56	6.46 ^a^	0.28	93.75 ^a^	1.06
10	0	1082.00 ^ef^	32.52	422.00 ^c^	19.79	660.00 ^ab^	12.72	1024.00 ^f^	46.66	602.00 ^ed^	26.87	5.19 ^e^	0.00	76.12 ^a^	10.92
10	2	1172.00 ^ed^	11.31	595.00 ^c^	57.98	577.00 ^bc^	46.66	2363.00 ^d^	523.25	1768.00 ^cb^	465.27	5.56 ^bc^	0.14	89.50 ^a^	1.06
10	5	1370.50 ^c^	16.26	823.00 ^b^	2.82	547.50 ^bc^	13.43	3070.50 ^c^	3070.50	2247.50 ^b^	5.00	5.83 ^cd^	37.47	90.02 ^a^	0.04
20	0	1077.50 ^ef^	55.86	545.50 ^c^	31.81	532.00 ^bcd^	24.04	1154.00 ^ef^	52.32	608.50 ^ed^	20.50	5.29 ^ed^	0.04	86.32 ^ab^	1.16
20	2	1026.50 ^fg^	79.90	547.00 ^c^	39.59	479.50 ^cd^	40.30	1701.50 ^ed^	61.51	1154.50 ^cd^	21.92	5.33 ^ed^	0.00	88.77 ^a^	0.03
20	5	948.50 ^g^	6.36	554.00 ^c^	9.89	394.50 ^d^	16.26	1827.50 ^d^	27.57	1273.50 ^c^	37.47	5.36 ^ed^	0.04	0.04 ^a^	0.04

^1^ Different letters for a given dependent variable denote significant differences (α = 0.05) across treatment conditions for that independent variable. ^2^ SD is standard deviation. ^3^ Visc is viscosity. RVA is Rapid Visco Analyzer.

**Table 11:** Main effects on quality evaluation parameters ^1^.

Effect	Uniformity		Size		Thickness		Softness		Color	
	Mean	SD ^2^	Mean	SD	Mean	SD	Mean	SD	Mean	SD
DDGS (%)										
0	7.96 ^b^	0.66	7.30 ^a^	0.74	7.10 ^a^	0.99	8.36 ^a^	0.99	8.30 ^a^	0.74
10	8.60 ^a^	0.77	5.66 ^b^	1.34	6.23 ^b^	1.30	4.86 ^c^	1.27	7.70 ^b^	0.53
20	8.50 ^a^	0.93	7.20 ^a^	0.84	6.90 ^a^	1.24	6.93 ^b^	1.25	6.93 ^c^	0.78
SSL (%)										
0	8.56 ^a^	1.04	6.50 ^b^	1.50	6.96 ^a^	0.96	6.53 ^b^	1.96	7.50 ^a^	0.820
2	8.40 ^ab^	0.77	7.26 ^a^	0.04	6.44 ^a^	1.66	6.33 ^b^	1.62	7.63 ^a^	1.88
5	8.10 ^b^	0.60	6.40 ^b^	1.10	6.80 ^a^	1.39	7.26 ^a^	1.91	7.80 ^a^	0.55

^1^ Different letters for a given dependent variable denote significant differences (α = 0.05) across treatment conditions for that independent variable. ^2^ SD is standard deviation.

**Table 12:** Treatment effects on quality evaluation parameters ^1^.

DDGS (%)	SSL (%)	Uniformity		Size		Thickness		Softness		Color	
		Mean	SD ^2^	Mean	SD	Mean	SD	Mean	SD	Mean	SD
0	0	7.70 ^d^	0.82	7.10 ^ab^	0.87	7.00 ^b^	0.94	8.50 ^ab^	0.89	8.10 ^b^	0.73
0	0	7.90 ^d^	0.56	7.70 ^a^	0.82	7.50 ^ab^	1.26	7.80 ^bc^	0.918	8.80 ^a^	0.78
0	0	8.30 ^bcd^	0.48	7.10 ^ab^	0.31	6.80 ^bc^	0.63	8.80 ^a^	1.03	8.00 ^bc^	0.41
10	2	9.10 ^a^	0.87	4.90 ^c^	1.97	7.00 ^b^	1.05	4.20 ^e^	0.42	7.70 ^bc^	0.48
10	2	8.60 ^abc^	0.51	6.70 ^b^	1.05	6.10 ^cd^	0.87	5.40 ^d^	1.64	7.50 ^c^	0.70
10	2	8.10 ^cd^	0.56	5.4 ^c^	1.17	5.60 ^d^	1.57	5.00 ^ed^	1.24	7.90 ^bc^	0.31
20	5	8.90 ^ab^	0.87	7.50 ^ab^	0.84	6.90 ^bc^	0.94	6.90 ^c^	0.99	6.70 ^d^	0.48
20	5	8.70 ^abc^	0.94	7.40 ^ab^	0.69	5.80 ^d^	0.42	5.90 ^d^	1.19	6.60 ^d^	0.84
20	5	7.90 ^d^	0.73	6.70 ^b^	0.82	8.00 ^a^	0.47	8.00 ^ab^	0.47	7.50 ^c^	0.70

^1^ Different letters for a given dependent variable denote significant differences (α = 0.05) across treatment conditions for that independent variable. ^2^ SD is standard deviation.
